# Primers for fourteen protein-coding genes and the deep phylogeny of the true yeasts

**DOI:** 10.1111/1567-1364.12059

**Published:** 2013-07-15

**Authors:** Vassiliki Koufopanou, Jonathan Swire, Susan Lomas, Austin Burt

**Affiliations:** Department of Life Sciences, Imperial College LondonAscot, UK

**Keywords:** *Saccharomycetales*, PCR primers, phylogenetics

## Abstract

The *Saccharomycetales* or ‘true yeasts’ consist of more than 800 described species, including many of scientific, medical and commercial importance. Considerable progress has been made in determining the phylogenetic relationships of these species, largely based on rDNA sequences, but many nodes for early-diverging lineages cannot be resolved with rDNA alone. rDNA is also not ideal for delineating recently diverged species. From published full-genome sequence data, we have identified 14 regions of protein-coding genes that can be PCR-amplified in a large proportion of a diverse collection of 25 yeast species using degenerate primers. Phylogenetic analysis of the sequences thus obtained reveals a well-resolved phylogeny of the *Saccharomycetales* with many branches having high bootstrap support. Analysis of published sequences from the *Saccharomyces paradoxus* species complex shows that these protein-coding gene fragments are also informative about genealogical relationships amongst closely related strains. Our set of protein-coding gene fragments is therefore suitable for analysing both ancient and recent evolutionary relationships amongst yeasts.

## Introduction

Yeasts are fungi that reproduce asexually by budding or fission, resulting in growth comprised mostly of single cells, and that have sexual states that are not enclosed within or formed upon a fruiting body (Kurtzman *et al*., 2011a). As yeasts are morphologically simple, our understanding of their phylogenetic relatedness to each other and to other fungi has been derived almost entirely from DNA sequence analysis. It is now clear that yeasts are found on several branches of the fungal tree of life, in both the *Ascomycota* and the *Basidiomycota* (Kurtzman *et al*., 2011c). The greatest number of described species (> 800) falls on a single branch in the *Ascomycota*, in the class *Saccharomycetes*, order *Saccharomycetales* (Kurtzman, 2011), sometimes also called the hemiascomycetes, or the ‘true yeasts’. This group includes the bakers’ and brewers’ yeast and model organism *Saccharomyces cerevisiae*, the human commensals and opportunistic pathogens *Candida albicans* and relatives, and numerous species used in biotechnology and involved in food spoilage (Cooper, 2011; Fleet, 2011; Johnson & Echavarri-Erasun, 2011).

The most widely used genes for phylogenetic analysis of yeasts are those coding for ribosomal RNA molecules, because they include regions that are highly conserved, allowing for PCR amplification from diverse species with a common set of primers, separated by regions that are more variable and phylogenetically informative (Kurtzman, 2011; Kurtzman *et al*., 2011b). However, the amount of phylogenetic information in rDNA has limits and is insufficient for delineating many early-diverging relationships (at least with current methods of analysis), with the result that the deep phylogeny of the *Saccharomycetales* is largely unresolved (Kurtzman, 2011). For closely related species, analysis of rDNA alone can also be problematic, because few, if any, nucleotide sites change as fast as neutrally evolving DNA and because the sequences only reflect the ancestry of a single region of the genome, whereas species delineation is best done using multiple unlinked genes (e.g. Koufopanou *et al*., 1997, 2006). There may also be evolutionary complexities associated with the fact that rDNA is a multicopy repeat (i.e. unequal crossing-over, nonallelic gene conversion and within-individual sequence variation), which can introduce uncertainties into genealogical interpretations (e.g. Poczai & Hyvönen, 2010).

Advances in technology mean that it is becoming increasingly feasible to sequence the complete genome of a yeast species, and such data will in future inform much of our understanding of yeast relationships (Medina *et al*., 2011). However, full-genome sequences are currently available for only a relatively restricted sample of species (predominantly members of the *Saccharomycetaceae* and *Debaryomycetaceae*; Dujon, 2010). Moreover, it may be some time before full-genome sequencing and analysis becomes easier and cheaper than multilocus PCR and sequencing for analysing large collections of isolates from closely related and cryptic species.

For these reasons, it is worthwhile expanding the set of targets available for PCR and sequencing, in particular to protein-coding genes. A few such genes, including translation elongation factor-1α and cytochrome oxidase II, have been used in some phylogenetic analyses (e.g. Belloch *et al*., 2000; Kurtzman & Robnett, 2003; Tsui *et al*., 2008; Kurtzman, 2011). Most recently, Kurtzman and Robnett (2013) have constructed a phylogeny of the type species of most described genera in the *Saccharomycetales* based on sequences of rDNA, translation elongation factor 1α and RNA polymerase II, subunits 1 and 2. The addition of these protein-coding genes substantially increased the resolution of the phylogeny. To further expand the array of protein-coding genes available for phylogenetic analysis, we have searched through published full-genome sequences for PCR-able regions and identified those that can be successfully amplified in a diverse array of *Saccharomycetales* yeasts. We have then sequenced these regions and used the data to infer phylogenetic relationships. We have also analysed published genomic sequence data from numerous strains in the *Saccharomyces paradoxus* species complex to assess how variable the fragments are amongst closely related lineages. We find that our set of protein-coding gene fragments is suitable for analysing both ancient and recent evolutionary relationships.

## Materials and methods

To identify promising gene fragments for phylogenetic analysis, we first downloaded published genomes for four ascomycete species *(Saccharomyces cerevisiae, Candida albicans, Neurospora crassa* and *Schizosaccharomyces pombe)* and performed a series of informatics searches for reciprocal best matches to identify putatively orthologous genes. First, each gene in *S. pombe* was aligned with every gene in *C. albicans*, using the Fasta3 local alignment algorithm with default parameters (Pearson, 2000), and the best match was recorded (i.e. the gene giving the highest *Z* score). This *C. albicans* gene was then aligned to every gene in the *S. cerevisiae* genome, and again, the best match was found. This *S. cerevisiae* sequence was then aligned to every gene in the *S. pombe* genome, and if the best match was the same as the starting *S. pombe* gene, then we classified the three genes as orthologs. For those genes passing this ‘triple boomerang test’, we also tested whether the *S. cerevisiae* gene had a reciprocal best match with an *N. crassa* gene. Together, these analyses allowed us to identify 2332 quartets of putatively orthologous proteins. To reduce the likelihood of including paralogs, we also recorded the second highest *Z* score for each of these searches and then excluded any gene for which this score was greater than 40% of the highest score or was itself higher than 156. This procedure gave 500 quartets for which the probability of including paralogs was relatively low.

Multiple alignment of the putative orthologs was performed with ClustalW, using default parameters (Thompson *et al*., 1994). Each alignment was then searched for candidate PCR primer binding sites, as follows. First, we recorded the maximum number of bases identical in all four organisms in a sliding window 20 nucleotides long. Then, we recorded the maximum separation in nucleotides of two potential primer sites in both of which at least *n*/20 bases were identical in all four organisms, for 12 ≤ *n* ≤ 18. The resulting lists of candidate gene fragments were then sifted manually to find those of an appropriate length (350–850 bp). The 28 most promising coding regions were then identified as being from the following genes: *GLN1, DRS2, CDC60, CDC19, FAS2, RPA135, SAH1, ATP2, PGK1, GLT1* (2 regions)*, GCD11, FBA1, PGI1, CRM1, RPO21, PDA1, ADE6, ATP1, VMA2, POL2, MET6, FAS1, ARG5, ARG6, ECM17, HTS1, NHP6A* and *GCV2* (gene names as used in *S. cerevisiae;* http://www.yeastgenome.org/). Degenerate PCR primers were designed for each of the 28 coding regions and tested on a set of 25 yeasts, chosen to represent a broad spectrum of yeast diversity (i.e. deep nodes in the 26S rRNA gene study of Kurtzman & Robnett, 1998; Table 1). Primers had between two and four degenerate sites to allow for differences between the four sequences, including usually no more than two inosine bases.

**Table 1:** Species used for PCRs and sequencing

					Genes sequenced†													
Clade	Code	CBS ID*	Species	No. of gene fragments sequenced	SA	GI	PG	DE	ME	AT	GC	FS	PA	VM	EC	OL	L T 1	L T 2
A	C31	6740^T^	*Lipomyces (Babjevia) anomalus*	9	1	1	0	1	1	1	0	0	1	0	1	0	1	1
	C38	7251^T^	*Lipomyces (Zygozyma) suomiensis*	10	1	1	0	1	1	1	0	1	1	1	0	1	0	1
	C17	2514^T^	*Lipomyces kononenkoae*	7	1	0	0	0	0	1	0	0	0	1	1	1	1	1
B	C30	6739^T^	*Candida sorbophila*	11	1	1	1	1	1	1	0	1	1	0	1	0	1	1
	C04	521.75^T^	*Blastobotrys aristata*	8	1	1	1	0	0	1	0	0	1	1	0	1	1	0
	C19	2594^T^	*Nadsonia fulvescens*	10	1	1	1	1	0	0	1	1	1	1	1	0	1	0
	C10	765.70^T^	*Dipodascus tetrasperma*	12	1	1	1	0	1	1	0	1	1	1	1	1	1	1
	C09	749.85^T^	*Dipodascus ambrosiae*	7	1	1	0	0	1	1	0	0	0	1	1	0	1	0
	C12	817.71^T^	*Dipodascus armillariae*	7	1	0	0	0	0	1	1	0	0	1	1	0	1	1
	C01	179.60^T^	*Galactomyces reessii*	11	0	1	1	1	1	1	0	1	0	1	1	1	1	1
D	C40	8139^T^	*Dekkera anomala*	4	0	0	1	1	0	1	0	0	0	0	0	0	1	0
	C37	7119^T^	*Pichia deserticola*	10	0	1	0	0	1	0	1	1	1	1	1	1	1	1
	C32	6929^T^	*Pichia pseudocactophila*	13	1	1	1	1	1	1	1	1	1	0	1	1	1	1
	C39	8071^T^	*Ogataea (Williopsis) salicorniae*	9	1	1	1	0	0	0	1	0	1	1	0	1	1	1
	C20	4111^T^	*Ambrosiozyma platypodis*	13	0	1	1	1	1	1	1	1	1	1	1	1	1	1
E	C21	4140^T^	*Nakazawaea (Pichia) holstii*	14	1	1	1	1	1	1	1	1	1	1	1	1	1	1
	C15	2286^T^	*Peterozyma (Pichia) xylosa*	12	1	1	1	0	1	1	1	1	1	1	1	0	1	1
F	C18	2555^T^	*Saccharomycopsis javanensis*	10	0	0	1	1	1	1	0	1	1	1	0	1	1	1
G	C34	6986^T^	*Wickerhamomyces (Pichia) alni*	13	1	1	1	0	1	1	1	1	1	1	1	1	1	1
	C36	7111^T^	*Phaffomyces (Pichia) antillensis*	11	1	1	1	0	1	1	0	1	1	1	1	1	1	0
	C23	5456^T^	*Barnettozyma (Pichia) salicaria*	13	1	1	1	1	1	1	1	1	1	1	1	1	1	0
	C33	6940^T^	*Starmera (Pichia) amethionina*	11	1	1	1	1	1	1	0	1	1	1	0	1	1	0
	C35	7023^T^	*Cyberlindnera (Lindnera, Pichia) mississippiensis*	11	1	1	1	0	1	1	1	1	1	0	1	1	1	0
	C02	254^T^	*Cyberlindnera (Lindnera, Williopsis) saturnus*	11	1	1	1	0	1	1	1	1	0	1	1	1	1	0
H	C03	398^T^	*Kazachstania (Saccharomyces) unisporus*	10	1	1	1	1	0	1	0	1	0	1	1	1	1	0

* T – type strain.

† Gene codes as in Table 2. 0 – not sequenced; 1 – sequenced. GenBank accession numbers KF042614-KF042824 and KF111756-KF111799.

Yeast strains were obtained from the CBS culture collection (Table 1). The species identity of all strains was confirmed by sequencing the D1/D2 region of the LSU rDNA (Kurtzman & Robnett, 1998). Yeast strains were grown on YPD plates (1% yeast extract, 2% peptone, 2% glucose) and DNA extracted following the protocol of Sherman (1991). Gene fragments were PCR-amplified in 50-μL reaction mixtures (Sigma-Aldrich REDTaq ReadyMix and 5 μL of a 10X dilution of template DNA), using 35 cycles of 95/50–60/72 °C for 30/60/120s, respectively. All primer pairs were tested first with an annealing temperature of 57 °C and then with a higher temperature (60 °C) if multiple bands were obtained at 57°, or with a lower temperature (50 or 55 °C) if no band was obtained at 57°. PCR products were visualised on agarose gels. From these initial PCRs, we chose 14 gene fragments for sequencing and phylogenetic analysis, based upon there being a single amplicon of approximately equal size from a substantial number of the test species (Table 2). Gene fragments were still accepted if some species had a single larger band, due to the likelihood that the extra length represented an intron. Primer sequences and alternate annealing temperatures for the 14 gene fragments are given in Table 3.

**Table 2:** Genes used for phylogenetic analyses

Code	Gene	Description	Number of species*	Length of alignment (amino acids)	No. of parsimony-informative characters	Min. possible no. of changes
SA	*SAH1*	Homocysteine hydrolase	38	279	84	271
GI	*PGI1*	Phosphoglucose isomerase	39	182	85	306
PG	*PGK1*	Phosphoglycerate kinase	36	268	97	391
DE	*ADE6*	Formylglycinamidine ribonucleotide synthetase	31	158	100	362
ME	*MET6*	Methionine synthase	36	160	65	208
AT	*ATP2*	ATP synthase	40	223	45	160
GC	*GCD11*	Translation initiation factor	30	219	79	264
FS	*FAS1*	Fatty acid synthetase	36	121	61	230
PA	*PDA1*	Pyruvate dehydrogenase	35	125	51	159
VM	*VMA2*	ATPase	38	180	28	67
EC	*ECM17*	Sulphite reductase	37	197	117	484
OL	*POL2*	DNA polymerase	36	150	83	327
LT1	*GLT1*	Glutamate synthase	42	149	89	417
LT2	*GLT1*	Glutamate synthase	32	176	103	369
18S	18S	18S rRNA gene†	30	1993	336	906

* Includes published genomic data from 18 species (17 for *PGK1* and *GLT1-2*).

† From GenBank; length of alignment measured in nucleotides, not amino acids.

**Table 3:** Primers used to amplify the 14 gene fragments

Name*	Sequence†	AAT (°C)‡
SA-1	G CAC ATG ACC AT**I** CA**R** AC**Y** GC	60
SA-4	CC GGT **I**GC GCA **I**CC **M**A**R** GTT	
GI-1	GAT TTG GG**I** CC**I** GT**Y** ATG GT	55
GI-2	TG TTG **I**A**I** GTA **R**GC **W**GG GAA	
PG-1	TC AG**I** GT**Y** GAC TTC AAC GTC CC	55
PG-2	GAA **I**GT GAA **R**GC CAT ACC ACC	
DE-1	CAC GAT GT**I** GGT GCT GGT GG	60
DE-2	GC AAC **I**GG **I**AC **Y**TG CCA AGG	
ME-1	C GAT ATG GTT CA**I** TAC TT**Y** GGT GA	50
ME-2	AT GGA AA**S I**AC **I**TC **R**GC ATC	
AT-1	GCT ATG GA**I** GGT AC**I** GA**R** GG	55
AT-2	AC GGC AGA **K**GG AAT ACG ACC	
GC-1	ATC GG**I** CA**I** GT**M** GCC CAC GG	–
GC-2	GA ACC ACC **I**GC AAC ACC **W**CC	
FS-1	CAA GA**I** CA**R** GG**Y** ATG GGT ATG GA	50
FS-2	TC ACC **I**A**I** AGA **R**TG ACC AGC	
PA-1	GGT AAG GGT GG**I** TC**I** ATG CA	–
PA-2	AC AGA CAT **I**GA **I**TG **R**CC ACC	
VM-1	AAG G**I**T GT**I** CA**Y** GAT GGT CA	50
VM-2	GT CAT **I**CC **I**TC ACC **R**AT **R**GC	
EC-1	G**YY** GGT GGT GGT ATG GG	–
EC-2	GG **R**CA ACC AGT CAT **I**C**K** CAT	
OL-1	GAT GT**I** GC**S** TC**I** ATG TA**Y** CC	50
OL-2	GC CAT TTC **I**AT **S**GA **R**TA CCA	
LT-1	ATG GA**R** CC**I** TGG GAT GG**Y** CC	55
LT-4	GG **I**GG ATT **I**GT **S**AC **Y**TG AGC	
LT-5	GCA CC**I** TG**Y** GA**I** GG**I** GCT TG	50
LT-6	CA ATC **I**TT ACC **I**GT **R**TC ACC	

* Odd numbers indicate forward primers; even numbers indicate reverse.

† I: inosine; otherwise IUPAC codes: R: A/G; W: A/T; M: C/A; K: T/G; Y: T/C; S: C/G.

‡ AAT: alternative annealing temperature. All primers were tested first at 57 °C, and then, most were tested at a second temperature, indicated here.

For sequencing, bands were cut out from the gel to remove the PCR primers, and the DNA was purified using Qiagen QIAquick columns and then sequenced using an AB 3700 capillary sequencer, following the manufacturer's instructions. DNA sequences were translated to amino acids to facilitate alignment and aligned using Clustal (Thompson *et al*., 1994). For phylogenetic analyses, we also included data from the orthologous regions of 13 yeast species with published genome sequences (*Candida albicans, C. glabrata, C. tropicalis, Clavispora lusitaniae, Debaryomyces hansenii, Eremothecium (Ashbya) gossypii, Kluyveromyces lactis, Lachancea (Kluyveromyces) waltii, Meyerozyma (Pichia) guilliermondii, Naumovozyma (Saccharomyces) castellii, Saccharomyces cerevisiae, Lachancea (Saccharomyces) kluyveri* and *Yarrowia lipolytica*) and five other ascomycete fungi, as outgroups (*Schizosaccharomyces pombe, Aspergillus fumigatus, A. nidulans, Neurospora crassa* and *Gibberella zeae*). Alignments were manually adjusted using MacClade (version 4.08; Maddison & Maddison, 2000), with regions of uncertain alignment excluded from subsequent analyses.

Phylogenetic analyses were performed on amino acid sequences rather than DNA sequences because yeasts show codon usage bias (Lloyd & Sharp, 1992), and differences between species in the strength or direction of this bias would make changes at different silent sites nonindependent. Phylogenetic analyses were conducted using PAUP* (version 4.0a122; Swofford, 2003), with alignment gaps (including missing genes) treated as missing data. First, a maximum parsimony search was performed (100 random additions, TBR branch swapping). The most parsimonious tree was then used in a likelihood analysis, which determined that the LG amino acid substitution rate matrix (Le & Gascuel, 2008) performed best, with amongst-site heterogeneity in substitution rates characterised by a discretised gamma distribution with a shape parameter equal to 0.75 (based on four categories) and a proportion of invariant sites equal to 0.31. A heuristic search was then performed using the LG matrix, empirical amino acid frequencies and these two parameter values, with 200 random additions, without branch swapping. The best 10 of these were then used as starting points for TBR branch swapping, with a reconnection limit of 8. Bootstrap support values were derived from 100 replicates, each involving 20 random additions, with NNI swapping. For parsimony analysis, the bootstrap supports are based on 100 replicates, each involving 100 random additions with TBR branch swapping.

To examine polymorphism of the 14 gene fragments amongst *S. paradoxus* strains, we used data from the Saccharomyces Genome Resequencing Project (Liti *et al*., 2009). Low-coverage genome sequences are available for 27 *S. paradoxus* strains (http://www.sanger.ac.uk/research/projects/genomeinformatics/sgrp.html). We extracted the part of the genomic alignment corresponding to each of our 14 gene fragments for analysis. As most of the genomes were sequenced with relatively low coverage, data were not available for all strains for all gene fragments. Bases identified in the database as having accuracy less than Q40 (i.e. expected error rate >10^−4^) were treated as missing data. Data were analysed at the DNA level as most of the variation is not expected to change the amino acid sequence.

## Results and discussion

### Identification and characterisation of suitable gene fragments

Our initial informatics and PCR analyses identified 14 primer pairs that amplified a single product from a large number of the 25 test species, which had been chosen to represent a broad spectrum of yeast diversity. Each of the 14 gene fragments was sequenced in an average of 18 species (range 12–24; Table 2), and each species was sequenced for an average of 10 gene fragments (range 4–14; Table 1). Overall, we obtained 257 sequences, 73% of the 14 × 25 = 350 possible. For the phylogenetic analyses, we included data from the orthologous regions of 18 published genome sequences; including these data brought the total number of protein-coding sequences analysed up to 506, 84% of the 14 × 43 = 602 possible.

The 14 gene fragments differ substantially in levels of variability, as measured by either the number of parsimony-informative characters or the minimum possible number of changes. On both counts, *ECM17* was the most variable fragment and *VMA2* the least variable (Table 2). The number of species analysed for each gene fragment varied from 32 to 42, but this number was not correlated with the observed variability of each gene. The combined data set had 2587 amino acid characters, of which 1087 were polymorphic and parsimony-informative, with a minimum of 4015 changes. For parsimony analyses, we also included 18S rRNA gene sequences, which were available for about half the species (Table 2), although the likelihood analyses used only the protein-coding sequences.

### Identification of major clades

**Figure 1:**
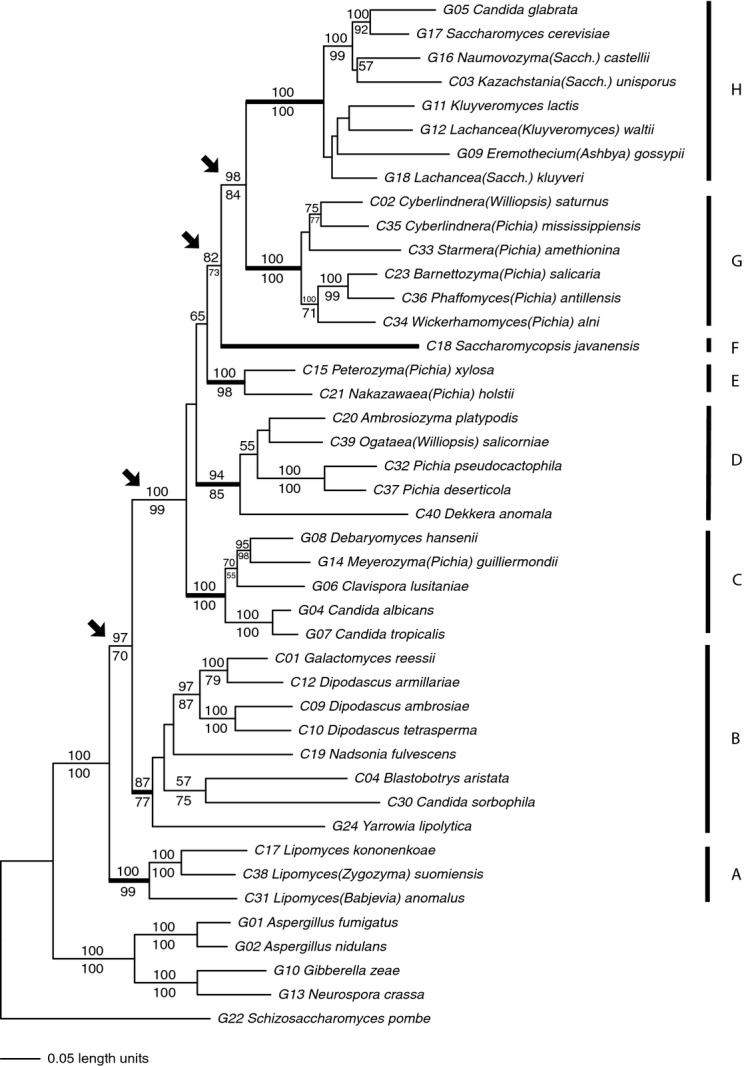
Phylogeny of 38 species of *Saccharomycetales* yeasts, plus five outgroups. Numbers above and below branches are bootstrap support statistics from likelihood and parsimony analyses, respectively. Branch lengths are proportional to inferred rates of evolutionary change. Thick lines in the phylogeny and lines and letters on the right indicate the main clades discussed in the text; arrows point to nodes showing the relationships amongst those clades.

The recent classification of the *Saccharomycetales* yeasts proposed by Kurtzman (2011) has 14 families and 21 genera *incertae sedis* (i.e. of uncertain placement). Our data set included representatives from nine of these families and six of these genera. Maximum-likelihood and parsimony analyses each gave well-supported phylogenies that agree well with each other, with bootstrap support values tending to be larger for the likelihood analysis (Fig. 1). In particular, each analysis identified eight well-supported clades, labelled A-H in Fig. 1. We will briefly describe the constituents of each clade and compare our results with those of Kurtzman and Robnett (2013). Except as noted below, it is difficult to identify morphological or physiological characters that distinguish the members of each clade from other yeasts.

#### Clade A (100/99% bootstrap support from likelihood/parsimony analyses, based on three species analysed)

This clade corresponds to the *Lipomycetaceae* (Kurtzman *et al*., 2007). Unifying characters for this family include similar cellular fatty acids and the production of extracellular starch-like compounds. Of the three species analysed, *Lipomyces kokonenkoae* clustered with *L. suomiensis*, to the exclusion of *L. anomalus*, in agreement with Kurtzman *et al*. (2007). Kurtzman and Robnett (2013) also found the *Lipomycetaceae* to be a clade (their clade 11).

#### Clade B (87/77% support with eight species)

This group contains a clade of four representatives from the *Dipodascaceae*, a clade of two species from the *Trichomonascaceae* and one species each from the genera *incertae sedis Nadsonia* and *Yarrowia*. This group had the weakest support of the eight main clades we identified, with most of the uncertainty due to *Y. lipolytica;* if this species was removed from the analysis, then the remaining species form a clade in 95% of parsimony bootstrap replicates. Kurtzman and Robnett (2013) also found a clade that includes the *Dipodascaceae*, *Trichomonascaceae* and *Yarrowia*, but without bootstrap support (their clade 9). Also included in their clade were three genera *incertae sedis* (*Magnusiomyces, Starmerella* and *Wickerhamiella*).

#### Clade C (100/100% support with five species)

This clade corresponds to the *Debaryomycetaceae* of Kurtzman & Suzuki (2010) and includes the well-studied human pathogen *Candida albicans* and relatives. This clade includes at least some species with a reassignment of the codon CUG from leucine to serine. It is difficult to unambiguously infer genetic code reassignments from our relatively short gene fragments, and so we did not include any species from this clade in the analysis except those for which there was a full-genome sequence. The relationship of species within this clade matched the relationships found by whole-genome analyses (Butler *et al*., 2009). The corresponding clade in Kurtzman and Robnett's (2013) analyses (their no. 6) also included members of the *Metschnikowiaceae* and *Cephaloascaceae*.

#### Clade D (94/85% support with five species)

This clade includes three members of the *Pichiaceae* plus one species each from the genera *incertae sedis Ambrosiozyma* and *Ogataea*. The most likely and most parsimonious trees showed these two genera nested within the *Pichiaceae*, although not with strong bootstrap support. Kurtzman and Robnett (2013) also found that these two genera formed a clade with the *Pichiaceae* (part of their clade 5).

#### Clade E (100/98% support with two species)

The two species in this clade are from the genera *incertae sedis Nakazawaea* and *Peterozyma*. Kurtzman and Robnett (2013) found that these two genera form a clade with another genus of *incertae sedis, Pachysolen*, and the three of these may in due course constitute a new taxonomic family.

#### Clade F (one species)

This clade contains the single member of the monogeneric family *Saccharomycopsidae* included in our analyses, *Saccharomycopsis javanensis*. Species in this genus are characterised by the abundant development of true mycelium and require that sulphur be available in organic form (e.g. from methionine; Lachance *et al*., 2000; Kurtzman & Smith 2011). Most species are facultative predators on other yeasts.

#### Clade G (100/100% support with six species)

This clade combines representatives of five genera, four of which had been placed by Kurtzman *et al*. (2008) in a new family, the *Wickerhamomycetaceae*, plus *Phaffomyces antillensis,* which had been placed in a different family (the *Phaffomycetaceae*; Kurtzman, 2011). The more recent sequence analysis has led to the suggestion that the *Wickerhamomycetaceae* appears to be a synonym of the earlier-described *Phaffomycetaceae* (Kurtzman & Robnett (2013; their clade 2), which our analysis also supports. In our analyses, *Phaffomyces antillensis* groups with strong bootstrap support with *Barnettozyma salicaria,* and these two species group together with *Wickerhamomyces alni* with strong bootstrap support, to the exclusion of the representatives from *Cyberlindnera* and *Starmera*.

#### Clade H (100/100% support with eight species)

This clade corresponds to the *Saccharomycetaceae*, which includes the well-studied *Saccharomyces cerevisiae* and relatives. Within this family, the four species descended from an ancient whole-genome duplication event grouped together with strong bootstrap support (i.e. *Candida glabrata* and the representatives from *Saccharomyces, Naumovozyma* and *Kazachstania*). Interestingly, our analysis also strongly supported the grouping of *C. glabrata* with *S. cerevisiae* to the exclusion of *N. castellii*. This arrangement is strongly supported by shared patterns of gene loss and other chromosomal rearrangements throughout the genome, although some whole-genome sequence analyses infer *C. glabrata* as the outgroup (Hess & Goldman, 2011; Rozpêdowska *et al*., 2011). Kurtzman and Robnett (2013) also found the *Saccharomycetaceae* to be a clade.

#### Compatibility of individual genes

It is interesting to know to what extent the results of the combined analysis outlined above are also found in separate analyses of each gene fragment. To address this question, we performed parsimony bootstrap analyses of each gene individually. As expected, none of the genes by itself was as informative as the combined analysis. Nevertheless, all of the clades A-H appeared on between two and eight of the individual bootstrap trees, except clade B, which did not appear on any of them (Table 4). Moreover, only one gene (*ATP2*) gave bootstrap support greater than 75% for a grouping that was incompatible with our identification of clades A-H, grouping together *Lipomyces (Babjevia) anomalus* and *Dekkera anomala* (76%). This grouping was not supported in bootstrap trees for any other gene.

**Table 4:** Bootstrap support (when > 50%) for clades A-H in individual gene analyses

	Clade							
Gene	A	B	C	D	E	F	G	H
*SAH1*					67	na	79	69
*PGI1*	59			96		na		
*PGK1*						na		
*ADE6*	62					na	77	
*MET6*	77					na	87	57
*ATP2*						na		
*GCD11*				98		na	95	
*FAS1*			91			na	97	
*PDA1*	89				74	na		96
*VMA2*						na		
*ECM17*				89	73	na	97	80
*POL2*	86					na	99	65
*GLT1-1*			94	68	65	na	80	93
*GLT1-2*	97			56	60	na		88

Parsimony bootstrap support for each of clades A-H in separate analyses of each gene. Note that in many cases, sequence data were not available for all members of the clade, but support was counted if the bootstrap analysis supported a clade with all the species for which there were data. na: not applicable: our clade F consists of only a single species in our data set and so is found by definition in all individual trees.

### Relationship amongst clades

Maximum-likelihood analysis gave strong support for the *Lipomycetaceae* (clade A) being sister group to the other yeasts, with 97% bootstrap support for a branch uniting the other yeast species together. Parsimony analysis also supported this placement, although less strongly (70%). The deepest divergence amongst the remaining yeasts was between clade B (the combination of the *Dipodascaceae*, the *Trichomonascaceae*, *Nadsonia* and *Yarrowia*) and the remainder, as clades C-H grouped together with 100% bootstrap support in maximum-likelihood analysis and 99% support in parsimony analysis. Finally, amongst the remaining clades, there was good support for clade G (the *Phaffomycetaceae*) being the sister clade of clade H (the *Saccharomycetaceae*; 98% and 84% bootstrap support from maximum-likelihood and parsimony analyses, respectively) and a link between these two clades and clade F (the *Saccharomycopsidae*; 82% and 73%). These findings are fully consistent with those of Kurtzman and Robnett (2013).

### Sequence variation amongst closely related strains

DNA sequences that are phylogenetically informative about ancient relationships may evolve too slowly to show variation amongst closely related strains. To assess the potential utility of our 14 gene fragments for population genetic and phylogeographic studies, we analysed published population genomic sequence data for the *Saccharomyces paradoxus* species complex (Liti *et al*., 2009). This complex consists of three genealogically independent lineages, in Europe, Far East Asia and America (Koufopanou *et al*., 2006). Average genomic sequence divergence of strains within each of the lineages is about 0.1%, whereas divergence between Europe and Far East Asian strains is about 1.4% and divergence between either one of these and American strains is about 4% (Bensasson *et al*., 2008; Tsai *et al*., 2008; Liti *et al*., 2009). Each of the gene fragments showed DNA sequence variation within and amongst the different lineages, with the number of polymorphic sites ranging from 7 to 23 (Table 5). The most variable gene fragments were *PDA1, GLT1-1, GLT1-2, SAH1* and *VMA2* (which was the least variable fragment in the *Saccharomycetales* amino acid data set); the least variable fragments were *PGK1, MET6, FAS1* and *PGI1*. Variability was not correlated with the number of isolates included in the analysis. As expected of such closely related sequences, parsimony analysis revealed little evidence of homoplasy (parallelisms, reversals or convergences): in all cases, the consistency index (CI) was 1, except for *PDA1* (CI = 0.958). Thus, every one of our gene fragments is polymorphic and contains information on genealogical relationships amongst closely related strains and thus could be used for population genetic or phylogeographic studies.

**Table 5:** Number of nucleotide sites in each of the gene fragments that are variable amongst strains of the *Saccharomyces paradoxus* species complex

Gene	No. of strains	No. of sites	No. of variable sites
*SAH1*	16	845	21
*PGI1*	14	580	9
*PGK1*	16	634	5
*ADE6*	26	488	12
*MET6*	12	482	7
*ATP2*	14	621	14
*GCD11*	19	665	16
*FAS1*	20	428	8
*PDA1*	19	451	23
*VMA2*	19	555	21
*ECM17*	17	664	12
*POL2*	19	614	18
*GLT1-1*	14	557	21
*GLT1-2*	17	544	21

For *PGI1*, there were no sequences available from the Far East Asia lineage; for all other genes, there were sequences available from at least one strain in each of the three *S. paradoxus* lineages.

## Conclusions

DNA sequence analysis has revolutionised our understanding of phylogenetic relationships amongst many taxa, microorganisms in particular. Much of this advance has come through the study of rDNA sequences, yet, useful as this gene cluster has been, other sources of information are needed to resolve many outstanding questions in reconstructing the tree of life. Protein-coding genes are an obvious source of such information, yet there has been the difficulty of extracting orthologous gene regions from diverse taxa using a common set of PCR primers. Primers that can amplify fragments of *TSR1* and *MCM7* from a wide variety of filamentous ascomycetes have recently been published (Schmitt *et al*., 2009). Our approach to this problem has been to scan published genome sequences for promising gene regions with well-conserved regions of about 20 bp, separated by a PCR-able length of 350–850 bp. We have identified 14 such gene regions, each of which can be amplified from more than half of a diverse set of *Saccharomycetales* yeast species using degenerate primers. The overall success rate was 73% and approximately the same success rate should be obtained with other diverse collections of *Saccharomycetales* yeast species. In some cases, failure to amplify appeared to vary consistently amongst clades (e.g. PG in clade A, LT2 in clade G); in other cases, it seemed more sporadic (Table 1). Slight modifications of the primer sequences or amplification conditions could potentially have increased further the success rate, but we did not consider this an efficient use of resources: the high bootstrap support levels we obtained suggested that we were already at the stage of diminishing returns.

In ‘supermatrix’ analyses such as presented here, in which data are combined across many genes, it is possible for maximum-likelihood analyses to give incorrectly strong support values when there is substantial and nonrandomly distributed missing data (Simmons, 2012). We do not think that this effect is likely in our analyses, as only 16% of gene sequences were missing, and all pairs of species had at least one gene sequence in common. In addition, parsimony analysis is apparently immune to this issue and found the same clades as the likelihood analysis. We chose to analyse amino acid sequences to avoid the potential problem of nonindependence due to differences in the strength or direction of codon usage biases. Compositional differences may still exist at the amino acid level, due, for example, to differences in amino acid synthesis costs or due to differences in the strength of biased gene conversion (Swire, 2007; Harrison & Charlesworth, 2011), and could be one source of error in our phylogenies. We have also assumed that all species use the universal genetic code, excepting the known CUG change in the *Debaryomycetaceae* (Santos *et al*., 2011).

Our phylogeny is strikingly concordant with that recently published by Kurtzman and Robnett (2013), despite the fact that completely different gene sequences were used (at least for our maximum-likelihood analysis); there was only partial overlap in the taxa included; and the methods of analysis differed (e.g. amino acid vs. nucleotide). This high degree of repeatability gives additional support to the relationships found and suggests that phylogenies can be reliably resolved with substantially fewer than the 50 genes suggested by Rokas *et al*. (2003). It is difficult to identify morphological features that distinguish the various clades from one another, indicating that such phenotypes (e.g. form of cell division, presence/absence of pseudohyphae and true hyphae, ascospore morphology) have been evolutionarily labile within the *Saccharomycetales*, with multiple parallelisms, convergences and/or reversals.

The primers and gene fragments identified in this paper can also be useful in studying closely related strains. Many named yeast species may, on closer analysis, be complexes of multiple cryptic biological species, and sequence-based methods for delineating them are best done by analysing multiple dispersed genomic regions, to test for genealogical concordance amongst loci (e.g. Koufopanou *et al*., 1997, 2006). The primers we have identified will be useful in analysing collections of closely related isolates that are more-or-less identical morphologically, metabolically and in their rDNA sequences, to determine whether they belong to one or more cryptic species. It would be a relatively simple matter to test the 14 primer pairs on a random sample of 5–10 isolates in the collection and then choose several (e.g. 5) of them that amplify most consistently for further analysis in the full collection. Such data will often allow one to delineate species boundaries and infer the rates and patterns of sexual reproduction and dispersal (Taylor & Fisher, 2003).

Only two of the four genomes used in designing the primer sequences were from the *Saccharomycetales*: the others were a filamentous ascomycete and a member of the early-diverging *Taphrinomycotina*. Our primers may work more broadly than just the *Saccharomycetales*, and it will be interesting to test how well they work in other ascomycetes and in Fungi more generally. The sequencing of selected protein-coding fragments may form a useful middle ground between rDNA and full-genome sequence analysis.
